# Scanning Electron Microscopy of Circulating Tumor Cells and Tumor-Derived Extracellular Vesicles

**DOI:** 10.3390/cancers10110416

**Published:** 2018-10-31

**Authors:** Afroditi Nanou, Mateus Crespo, Penny Flohr, Johann S. De Bono, Leon W. M. M. Terstappen

**Affiliations:** 1 Department of Medical Cell BioPhysics, University of Twente, 7522 NH Enschede, The Netherlands; 2 Division of Clinical Studies, The Institute of Cancer Research, London SM2 5NG, UK; Mateus.Crespo@icr.ac.uk (M.C.); Penny.Flohr@icr.ac.uk (P.F.); Johann.DeBono@icr.ac.uk (J.S.D.B.); 3 Prostate Cancer Targeted Therapy Group, The Royal Marsden NHS Foundation Trust, London SM2 5PT, UK

**Keywords:** scanning electron microscopy (SEM), circulating tumor cell (CTC), tumor-derived extracellular vesicle (tdEV), ferrofluid, CellSearch, microsieves

## Abstract

To explore morphological features of circulating tumor cells (CTCs) and tumor-derived extracellular vesicles (tdEVs), we developed a protocol for scanning electron microscopy (SEM) of CTCs and tdEVs. CTCs and tdEVs were isolated by immunomagnetic enrichment based on their Epithelial Cell Adhesion Molecule (EpCAM) expression or by physical separation through 5 μm microsieves from 7.5 mL of blood from Castration-Resistant Prostate Cancer (CRPC) patients. Protocols were optimized using blood samples of healthy donors spiked with PC3 and LNCaP cell lines. CTCs and tdEVs were identified among the enriched cells by fluorescence microscopy. The positions of DNA+, CK+, CD45− CTCs and DNA−, CK+, CD45− tdEVs on the CellSearch cartridges and microsieves were recorded. After gradual dehydration and chemical drying, the regions of interest were imaged by SEM. CellSearch CTCs retained their morphology revealing various shapes, some of which were clearly associated with CTCs undergoing apoptosis. The ferrofluid was clearly distinguishable, shielding major portions of all isolated objects. CTCs and leukocytes on microsieves were clearly visible, but revealed physical damage attributed to the physical forces that cells exhibit while entering one or multiple pores. tdEVs could not be identified on the microsieves as they passed through the pores. Insights on the underlying mechanism of each isolation technique could be obtained. Complete detailed morphological characteristics of CTCs are, however, masked by both techniques.

## 1. Introduction

Circulating Tumor Cells (CTCs) play a crucial role in the formation of metastases [1,2] and the CTC peripheral blood load is directly associated with the overall survival of cancer patients [3,4,5,6,7,8,9]. Many groups have focused their research on the development of different technologies [10,11,12,13] to increase capture efficiency of CTCs from blood samples. Understanding the biophysical features of CTCs by scanning electron microscopy (SEM) could contribute to the future improvement of the existing or the development of new CTC isolation techniques. A detailed morphological characterization of isolated CTCs with this technology has not been studied so far. SEM has been used to visualize cells from cancer cell lines mainly on microfluidics devices or filters [14,15]. SEM images of CTC clusters from patient samples have also been shown previously [16]. However, to our knowledge, cell lines captured after being spiked into blood samples of healthy donors as well as single CTCs from the blood of cancer patients have not been studied using SEM imaging. To obtain SEM images of CTCs, we chose to isolate CTCs from the blood of Castration-Resistant Prostate Cancer (CRPC) patients by physical separation and immunomagnetic selection. For both approaches, a preparation protocol for SEM imaging needed to be developed for the optimal morphological preservation of cells once in the vacuum. Therefore, preceding the SEM imaging of CTCs, the preparation protocol was first developed and optimized on cells from tumor cell lines spiked in blood samples of healthy donors that were EpCAM enriched using the CellSearch system or isolated based on their size and deformability by the passage of blood through 5 μm microsieves. The developed protocol was applied on the enriched samples from CRPC patients. 

In an earlier report, we showed that CTCs have a large range of morphological appearances and proved that a large portion of them are undergoing apoptosis [17]. In a later study, we subdivided them into morphological subclasses, including “intact CTC”, “tumor cell fragments”, and “tumor microparticles”, and showed that they all strongly correlated with clinical outcomes [18]. These EpCAM+, CK+, CD45− objects, baptized tumor-derived extracellular vesicles (tdEVs), can now be classified with objective criteria using the open-source image analysis software, Automatic CTC Classification, Enumeration and PhenoTyping (ACCEPT) (http://github.com/LeonieZ/ACCEPT), and are equivalent to CTCs in terms of prognosis of CRPC patients [19]. Here, we used SEM imaging to gain more insights in the morphological features of CTCs and tdEVs. 

## 2. Results

### 2.1. Cell Preparation for SEM Imaging

**Figure 1:**
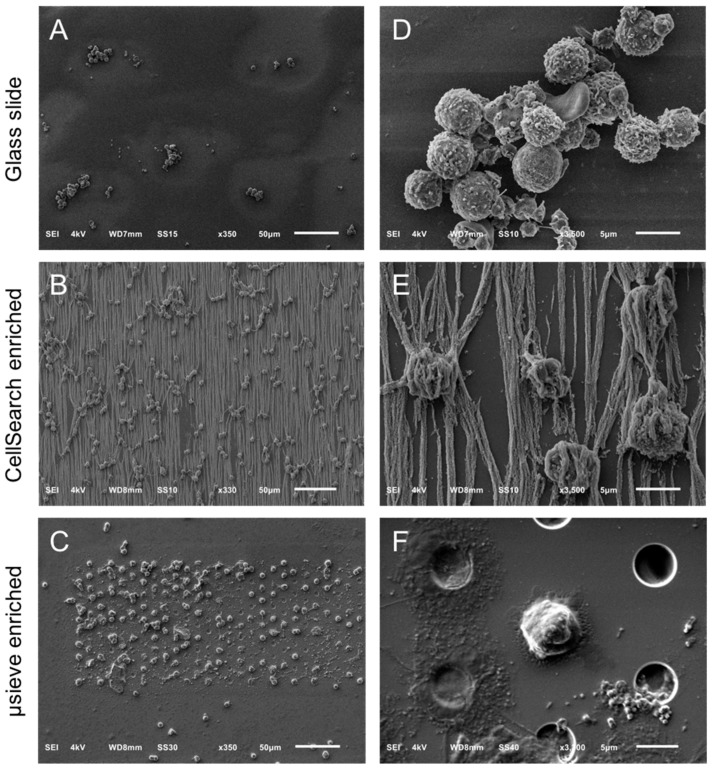
Overview of cells by SEM on a glass slide (Panel **A**), a CellSearch cartridge (Panel **B**) and a 5 μm pore microsieve (Panel **C**). A higher magnification of the isolated cells is shown at the right of each technique (Panels **D**–**F**). The vertical lines in panels **B** and **E** is the αEpCAM ferrofluid covering the surfaces of all isolated objects and are perfectly aligned with the magnetic field lines [20].

**Figure 2:**
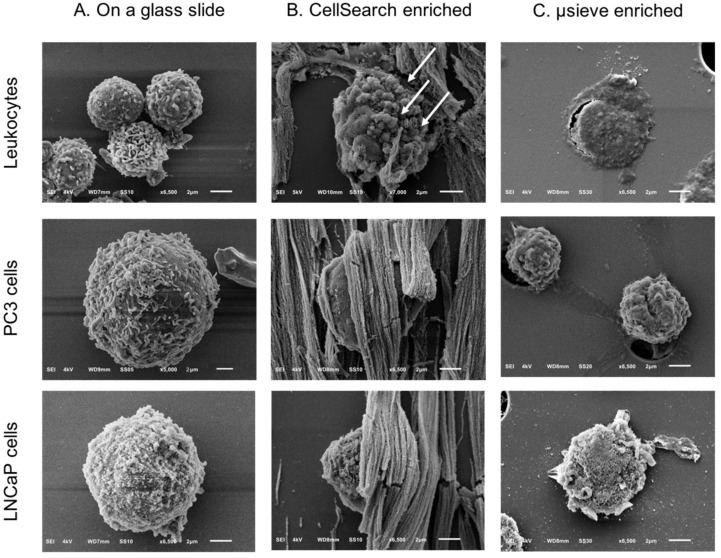
SEM images of leukocytes, PC3, and LNCaP cells on a glass slide (Panel **A**) and after being spiked in blood and isolated by the CellSearch (Panel **B**) and on 5 μm pore microsieves (Panel **C**). The arrows (Panel **B**) are pointing at spherical vesicles on the surface of a leukocyte isolated by the CellSearch.

A preparation protocol of cells for their subsequent SEM imaging was developed as described in Section 4.5. PC3 and LNCaP cells in suspension were used for the optimization of the protocol. A diagnostic leukapheresis (DLA) sample of a prostate cancer patient sample was used to obtain SEM images of the leukocytes (Figure 1, Panels A,D). The protocol preserved cell shape, morphology, and distinct surface features distinguishing leukocytes, PC3, and LNCaP cells (Figure 2, Panel A). The sizes of PC3 and LNCaP cells were much larger as compared to the sizes of leukocytes. 

### 2.2. Overview of Cells by SEM Imaging on Glass Slides, CellSearch Cartridges, and 5 μm Pore Microsieves

Figure 1 shows the SEM images of leukocytes on a glass slide (Panels A,D), after immunomagnetic enrichment on a CellSearch cartridge (Panels B,E) and on microsieves with 5 μm pores (Panels C,F). Panels A–C are shown in lower (330–350×) magnification and panels D–F at a higher (3300–3500×) magnification. Cells were found mainly in aggregates when prepared in cell suspension for SEM imaging; however, their surface features were very clear. On the other hand, the isolated cells on the CellSearch cartridge were in a monolayer and fully covered by the ferrofluid that is aligned according to the magnetic field lines [20]. Many cells on the microsieves were damaged. Some cells kept their spherical shape, without, however, keeping their cell surface features, probably due to the stress they experienced while entering the pores.

### 2.3. SEM Imaging of Isolated PC3 and LNCaP Cells Spiked in Blood by the CellSearch System and Microsieves

SEM images of PC3 and LNCaP cells in suspension were compared to the PC3 and LNCaP cells, which were isolated by CellSearch or on microsieves after being spiked in blood samples (Figure 2). In most of the cases, the PC3 and LNCaP cells that were isolated by CellSearch were fully covered by ferrofluid. The images shown in panel B of Figure 2 reveal small parts of their surfaces. In these minor exposed parts, PC3 and LNCaP cells seem to have retained their distinctive morphological features, with PC3 having more heterogeneous surface with very smooth portions and elongated microvilli in some parts whereas LNCaP was more homogeneous with microvilli covering all their surface. Nonetheless, in the case of microsieves, apart from few LNCaP cells that seemed to have some well-preserved their surface characteristics, most of the isolated cells lost their distinctive microvilli that were found when cells were in suspension. Moreover, many DNA+ cells after being relocated and SEM imaged, were flattened with surface defects, implying that their nucleus was inside the pores and only the membrane was left on the top. Other cells were within more than one pore, indicating that cells underwent a lot of stress during their filtration. Stripes connecting the neighboring pores could also be observed (Figure 2, Panel C, PC3 cells), implying the fragmentation of formerly passed cells through the 5 μm pore microsieves or the cell tearing of the imaged ones. 

### 2.4. Relocation and Correlated SEM-Fluorescence Images of CTCs and tdEVs of CRPC Patients Isolated by the CellSearch

**Figure 3:**
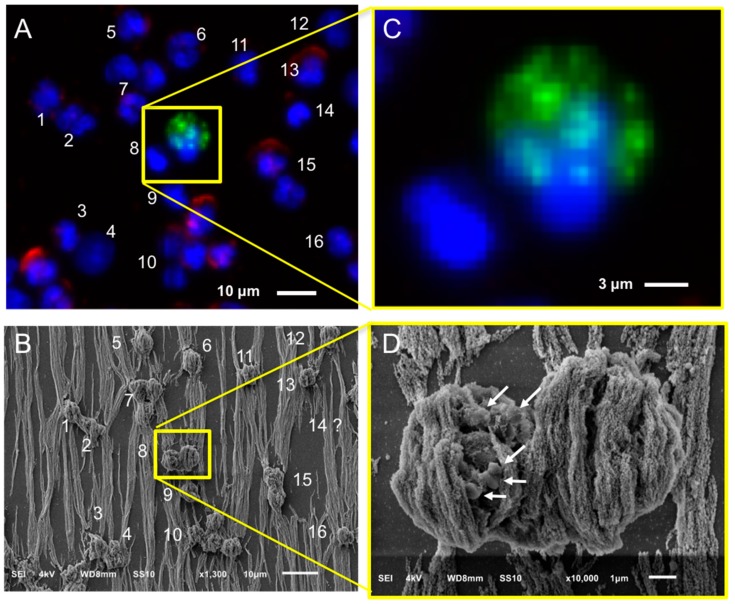
Relocation of an identified CTC of a CRPC patient by fluorescence (CellTracks Analyzer II) imaging (Panels **A** and **C**) after immunomagnetic enrichment using the CellSearch system and SEM imaging (Panels **B** and **D**). Magnified fluorescence and SEM images of the enclosed by the yellow square CD45−, CK− nucleated cell (left), and CD45−, CK+ apoptotic CTC (right) are shown at Panels **B** and **D**, respectively. Nucleus (DNA) is represented by blue, CK by green, and CD45 by red. The arrows of Panel **D** are pointing at vesicles on the surface of a cell of unknown lineage (DNA+, CK−, CD45−).

**Figure 4:**
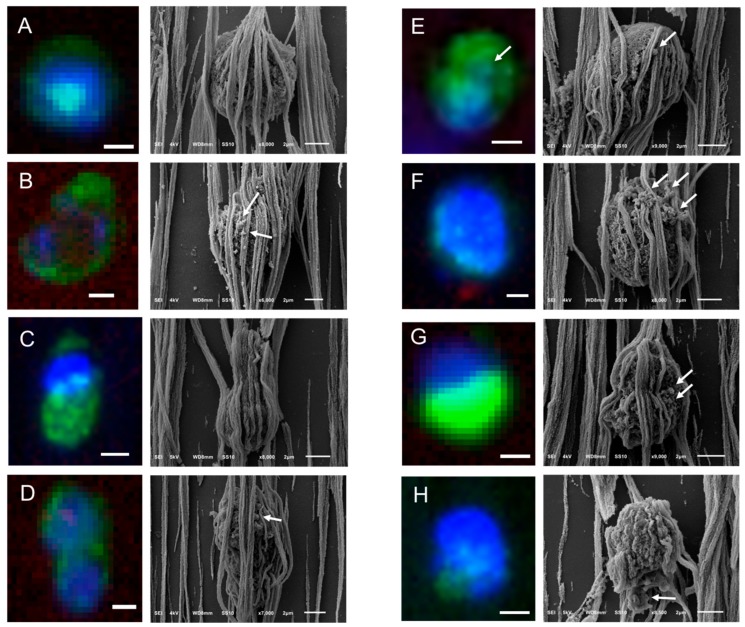
Gallery of correlated fluorescence and SEM images of CTCs of a CRPC patient isolated by the CellSearch system. Nucleus (DNA) is represented by blue and CK by green. The arrows are pointing at spherical vesicles on the surface of CTCs (Panels **D**–**G**), single or aggregated vesicles on top of the ferrofluid covering the CTCs (Panels **B**,**F**), and bigger CK+ particles or membrane fragments of a not well defined shape (Panel **H**). Scale bars on fluorescence images indicate 3 μm.

After immunomagnetic enrichment by CellSearch, all objects are covered by ferrofluids, shielding a major portion of the cell surfaces. Hence, to ensure which of the enriched cells were CTCs, the region of interest containing the CTCs from the fluorescence images were found using SEM. Figure 3 shows an example: In Panel A, one of the fluorescence images of a cartridge of a CRPC patient is shown, with nucleus (DNA) shown in blue, CD45 (membrane marker of leukocyte) shown in red, and cytokeratin CK (intracellular marker of epithelial cell) shown in green. The corresponding SEM image is shown in Panel B, and the position of individual cells in both fluorescent and SEM images is indicated with numbers. Sometimes, cells (#14 is an example) could not be relocated in the SEM image and were most likely removed through the dehydration/drying procedure. The yellow square in Panels A and B encloses two cells, one of which is clearly a CTC and the other one is of unknown lineage of origin as no CD45 or CK staining could be discerned. Both cells are shown at higher magnification in Panels C and D. The cytokeratin of the CTC shows a punctuated pattern that is characteristic for CTC undergoing apoptosis [17,21]. This punctuated pattern cannot be seen in the SEM image either because the CTC is covered by the ferrofluid or because this pattern is only intra-cytoplasmatic. The lineage of origin of the other cell is not known, the nucleus does not show the typical shape of a granulocyte that can be seen in other nuclei and there is no staining with CD45. Big vesicles at the surface, as indicated with the arrows in Panel D (Figure 3), were seen in 55% of the imaged leukocytes (47 out of 85) as shown in Panel A, Figure S5. 15% of leukocytes (13 out of 85) had smaller vesicles on their surfaces (Panel B, Figure S5) similar to the ones observed on 52% of the imaged CTCs (11 out of 21) we identified (Figure 4 and Figure S1). Hence, the finding of vesicles solely on the surface of an isolated cell without CK (or any other epithelial/tumor-specific) staining is not sufficient to suggest that this is indeed a CTC.

Following the same procedure, CTCs of CRPC patients identified by the CellSearch system were relocated using SEM and a gallery of correlated fluorescence and SEM images of CTCs was constructed (Figure 4 and Figure S1). 

Most of the CTCs have a comparable size to the leukocytes isolated by the CellSearch system as shown in Figure 3, supporting the previous findings of Ligthart et al. that 20–40% of CTCs in breast, prostate, and colorectal cancer patients have a diameter close to 10 μm similar to the diameter of leukocytes [22]. On the contrary, PC3 and LNCaP cells that were used as controls for the optimization of the protocols were clearly bigger than leukocytes and easily distinguishable from the rest of the leukocytes even when low magnification was used with SEM. 

Different shapes of CTCs were found in the CellSearch cartridges as shown in Figure 4 and Figure S1. 67% of CTCs (14 out of 21) were spheroid to ovoid (Panels C,D of Figure 3, Panels A,E,F,H of Figure 4, Panels A–C,E,F,H,J–L of Figure S1). Interestingly, 19% of CTCs (4 out of 21) bore a protrusion from one side (Panels B,C of Figure 4, Panel D of Figure S1) or from both sides, resembling a sigmoidal shape (Panel I, Figure S1), which was not expected from the respective fluorescence image. 19% of CTCs (4 out of 21) were eight-shaped (Panels C,D,G of Figure 4, Panel G of Figure S1) likely corresponding to different phases of a CTC cycle, such as telophase (Panel D of Figure 4) and anaphase (Panel G of Figure S1). On the contrary, PC3 and LNCaP cells (Panel A, Figure S4) appeared larger and more homogeneous with a spheroid to ovoid shape. 

The surface of CTCs appeared to be smooth and sponge-like because of some holes they seem to have on their surface regardless of their speckled cytokeratin, as denoted by arrows on Panels G,I,J of Figure S1. In addition, the 21 CTCs that were imaged from two different patients did not have distinctive microvilli as PC3 and LNCaP cells (Panels A,B of Figure 2, Panel A of Figure S4); it is expected that once tumor cells intravasate and circulate in the bloodstream, they undergo epithelial to mesenchymal transition (EMT), obtaining a more mesenchymal phenotype. During that process, it is expected that they will not need specific receptors to grow as while adherent.

In many cases, CTCs had vesicles, as indicated by arrows in Figure 3, Figure 4 and Figure S1. The CTC of Panel E, Figure 4 has a vesicle on its surface that corresponds to a cytokeratin speckle in the respective immunofluorescence image, implying that the secreted vesicles of CTCs express also cytokeratin. The ferrofluid covering all the cells, however, hinder their morphological characterization in depth.

In Panels B,C of Figure S1, the speckled cytokeratin pattern of the apoptotic CTCs corresponds to extra-cytoplasmatic vesicles. Some of these vesicles are clearly above the ferrofluid, raising questions about their origin. Are they vesicles extruded from the CTCs’ surface during their capture by the αEpCAM ferrofluid or vesicles not related to the specific CTCs that were present in the enriched cell suspension? The hypothesis that they are vesicles derived from the CTC can be supported by the observation that similar structures are seen on some CTCs found on the microsieves (Figure S2, Panels D,H,J,N,O,S,T). On the other hand, the fact that these particles were not only found in the close vicinity of the specific CTCs, but also spread in a part of the cartridge also covering leukocytes, would support the second hypothesis. However, both cases could be true.

### 2.5. Relocation and Correlated SEM-Fluorescence Images of tdEVs of CRPC Patients Isolated by the CellSearch

**Figure 5:**
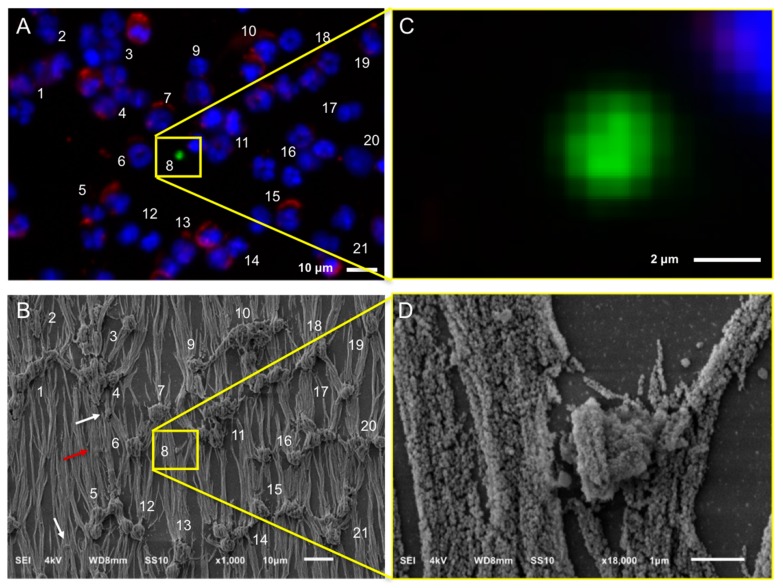
Relocation of an identified tdEV of a CRPC patient by fluorescence (CellTracks Analyzer II) imaging (Panels **A**,**C**) after immunomagnetic enrichment using the CellSearch system and SEM imaging (Panels **B**,**D**). Magnified fluorescence and SEM images of the enclosed by the yellow square DNA−, CK+, CD45− tdEV are shown at Panels **B**,**D**, respectively. Nucleus (DNA) is represented by blue, CK by green, and CD45 by red. The white arrows (Panel **B**) are pointing at vesicles of not defined origin (DNA−, CK−, CD45−). The red arrow (Panel **B**) is pointing at a leukocyte derived- extracellular vesicle (DNA−, CK−, CD45+).

**Figure 6:**
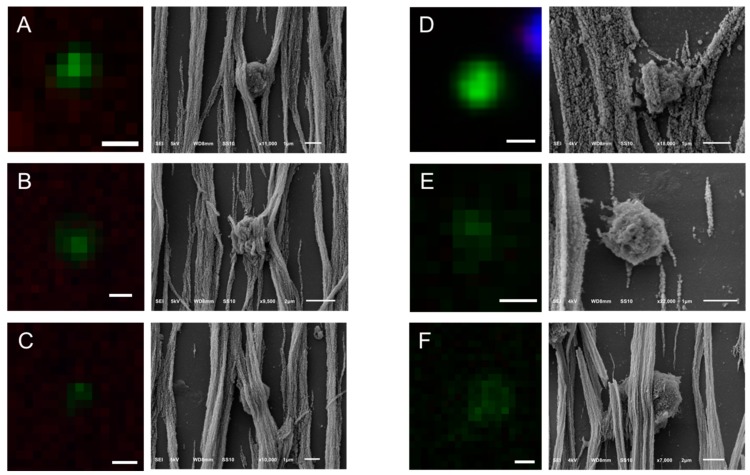
Gallery of correlated fluorescence and SEM images of tdEVs of a CRPC patient isolated by the CellSearch system. CK is represented by green. The pixels with lower CK intensity (Panels **A**–**C**,**F**) correspond to being shielded from ferrofluid tdEV parts. tdEVs of different CK intensities can be observed (Panel **D**: High CK intensity, Panels **A**–**C**: Medium CK intensity, Panels **E**,**F**: Low CK intensity). Scale bars of fluorescence images indicate 2 μm.

The same procedure that was described for the SEM imaging of the CTCs was followed in the case of EpCAM+ tdEVs isolated from the blood of CRPC patients by CellSearch. An example is shown in Figure 5. A DNA−, CK+, CD45− tdEV is surrounded by leukocytes (Panel A). The same tdEV can be hardly distinguished at 1000× magnification in the respective SEM image (Panel B) in contrast to the easily recognizable cells. The small size of tdEVs in combination with the ferrofluid covering them as well as the rest of the surface of the cartridge hinders their 3D shape and makes their relocation laborious. Due to that, a limited number of tdEVs (only six) were SEM imaged. However, as we have demonstrated previously, tdEVs can be found in significantly elevated numbers in CRPC patients (median value of 116 tdEVs in 84 CRPC patients) compared to healthy donors (median value of 8 tdEVs in 16 healthy donors) [19]. The correlated fluorescence and SEM images of tdEVs of CRPC patient blood samples are summarized in Figure 6. Isolated tdEVs are much smaller compared to leukocytes, nucleated cells, or CTCs, with the majority of them having a size range of 1–2 µm based on the SEM images of Figure 6. The shape of all tdEVs shown here is spheroid to ovoid; however, digitally stored fluorescence images of CRPC patients imply also a subpopulation of tdEVs with a tubular shape. However, this shape is less frequent (5–10% of the total tdEV population, data not shown). Different CK intensities can be recorded in the fluorescence images of tdEVs, with some of them having very dim CK signals (Panels E,F). Nevertheless, the SEM images confirm that they are not just image artefacts, but actual particles isolated by the blood samples of the patients in the same manner as CTCs as shown by the ferrofluid covering them. The parts of tdEVs that are shielded by the ferrofluid have lower CK signals, denoting that the ferrofluid is reducing the resulting fluorophore signals, which can be crucial in the case of small EVs to be detected by solely one biomarker.

A DNA-, CD45+ particle is indicated by a red arrow in Figure 5 (Panel B), suggesting that particles of leukocyte origin are co-isolated with the CellSearch system. Further investigation is required to answer the raised question of whether these particles are fragments as a result of the CellSearch procedure or actual extracellular vesicles (EVs) secreted by leukocytes. 

It is noteworthy to mention that more particles are suspected to be isolated by the CellSearch system that cannot be detected by fluorescence because either they do not express any CD45 or CK, or they express CD45 or CK at levels below the detection limits of our fluorescence microscopes. Some of these particles that have a very similar shape and morphology as the tdEV of Figure 5 are pointed out by white arrows in Figure 5 (Panel B); however, the highlighted particles were not SEM imaged with a higher magnification. A higher magnification of a different particle undetectable by the CellTracks Analyzer II (DNA−, CD45−, CK−) was SEM imaged and can be found in Figure S3. 

### 2.6. Relocation and Correlated SEM-Fluorescence Images of CTCs of CRPC Patients Isolated by Microsieves

The recommended VyCAP protocol was first applied for the permeabilization, fixation, and staining of cells isolated by microsieves. However, that led to a lot of red blood cell background during their SEM imaging, most likely due to the fixation step that preceded the cell permeabilization step (Figure S6); hence, a different protocol that is similar to the protocol used by the CellSearch was eventually followed for both control and patient samples and is described in Section 4.4. 

**Figure 7:**
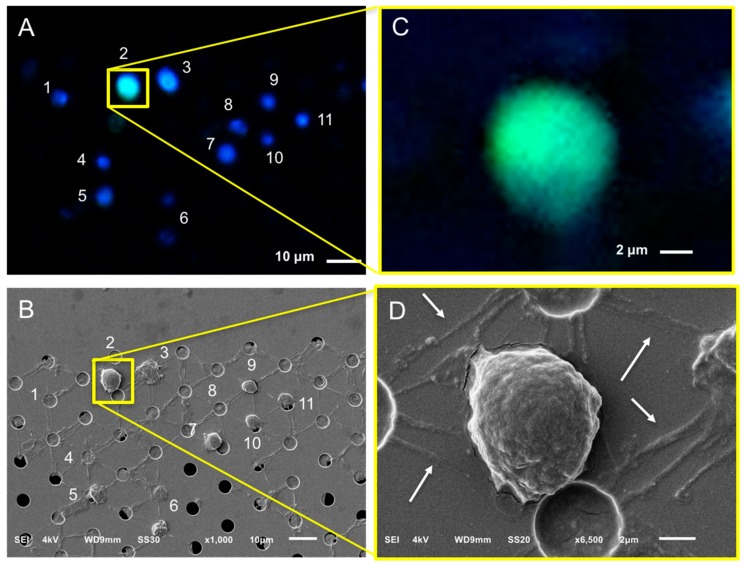
Relocation of an identified CTC in a blood sample of a castration-resistant prostate cancer patient after microsieve filtration and fluorescence imaging (Panels **A**,**C**). Pores were relocated and the respective cells were SEM imaged (Panels **B**,**D**). Nucleus (DNA) is represented by blue and CK by green. Fluorescence and SEM images of the enclosed by the yellow square DNA+, CK+, CD45− CTC at Panels **A**,**B** are shown with higher magnification at Panels **C**,**D**, respectively. Arrows (Panel **D**) are pointing at the membrane or/and nucleus stripes of cells connecting the neighboring pores.

Most spiked PC3 and LNCaP cells in blood, after being filtered through the microsieves, had lost their surface features (Figure 2 and Figure S4). The loss of surface features was tested in 20–30 different spiked cells (PC3 or LNCaP) in three replicates. Therefore, the relocation of identified DNA+, CK+, CD45− CTCs based on the fluorescence images had to be relocated using SEM (Figure 7). The procedure was less time consuming compared to SEM imaging of the CTCs in the CellSearch cartridges thanks to the specific pore locations of the microsieves. Cells in Figure 7 with bright DNA signals (#2,3,7,9–11) seemed to be more intact compared to the cells with dim DNA signals (#1,4,6), which appeared to be damaged or within the pores. 

**Figure 8:**
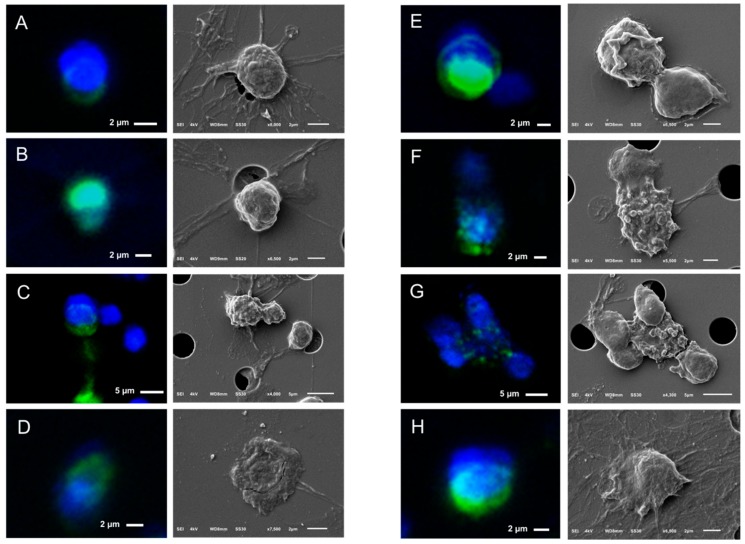
Gallery of correlated fluorescence and SEM images of CTCs of a CRPC patient isolated by whole blood filtration using 5 μm microsieves. Nucleus (DNA) is represented by blue and CK by green. Membrane (Panel **C**) or nucleus (Panel **B**) stripes connecting the neighboring pores indicate cell damage and were found throughout the whole microsieve. CTCs ended up on (Panels **A**–**C**,**F**,**H**), within (Panel **D**), or in between the pores (Panels **E**,**G**,**H**).

By relocating the CTCs on microsieves from the whole blood of one CRPC patient, we constructed a gallery of correlated fluorescence and SEM images of 28 CTCs shown in Figure 8 and Figure S2. CTCs were found on top, within, or between the pores of the microsieves.

The SEM images showed some stripes connecting the neighboring pores (indicated by arrows on Panel D of Figure 7), implying that the cells were split into more than one pore during their passage through the microsieves. These stripes were either cell membranes or nuclei as indicated by the respective immunofluorescence images of CTCs (Figure 7, Figure 8 and Figure S2). More specifically, the CK staining of Panel C of Figure 8 corresponds to the membrane of a CTC reaching the pore, whereas the DNA signal of panel B of Figure 8 and Panel C of Figure 7 corresponds to the fragmented nuclei of previously passed cells through the microsieves. In some cases, it is not clear whether these stripes originate from the cells occupying a pore or from previous cells that passed during blood filtering (Panels A,H of Figure 8).

Besides “intact” CTCs, apoptotic CTCs with a punctuated cytokeratin pattern were found on the microsieves (Panels F,G of Figure 8). The vesicles on their cell surface suggest that the CK punctuated pattern is not just intra-, but also extra- cytoplasmatic.

No distinctive surface characteristics, like microvilli, could be found on CTCs after filtration, which is similar to the PC3 and LNCaP cell lines that were used as a control. 

Regarding their shape, 100% of CTCs from a single patient (28 out of 28) appeared to be spheroid to ovoid, probably due to the filtration procedure. Tubular or different shaped CTCs either passed through the filters together with the smaller CTCs or appeared to be spherical once captured on the microsieves.

## 3. Discussion

CTC load is associated with relatively poor prognosis in patients with metastatic cancer, and can be used to monitor the efficiency of a therapy [5,6,7,8,9,23]. CTC data has been generated mainly by the CellSearch platform, but new technologies are emerging for CTC isolation [10,11,12,13]. Each technique has a different underlying principle, resulting in CTCs with selected characteristics. Consequently, the CTC identification can differ between the different existing techniques, raising the question whether phenotype differences matter. Size-based isolation techniques, like the microsieves, miss CTCs of sizes smaller than the selected pore diameter, whereas affinity-based techniques, such as the EpCAM-based CellSearch system, will miss the EpCAM- or EpCAM_low_ CTCs [24]. After immunomagnetic enrichment, the presence of not only CTCs with “intact” morphology, but also those undergoing apoptosis [25,26,27,28] as well as tumor-derived extracellular vesicles [18,19] are associated with poor clinical outcome. Here, we investigated by SEM imaging the morphology of intact and apoptotic CTCs isolated by the CellSearch system and 5 μm microsieves from whole blood of CRPC patients. tdEVs were possible to be SEM imaged only when the CellSearch platform was used; in the case of microsieves, they ended up in the filtrate.

CellSearch enriched CTCs, and tdEVs had a well retained morphology and shape, but their surfaces were fully covered by the ferrofluid, hindering the study of their surfaces. The complete cell coverage can be explained by the underlying principle of CellSearch. Briefly, αEpCAM ferrofluid is incubated in blood samples of cancer patients and binds to EpCAM+ CTCs and tdEVs. The ferrofluid together with the bound cells are pulled out of the tube into the CellSearch cartridge by the application of magnetic forces. The enriched objects in the cartridge are perfectly aligned to the magnetic field lines of the magnets [20]. The excess of ferrofluid could partially explain the dim or negative signal of cells when stained for additional surface biomarkers, like the prostate-specific membrane antigen (PSMA) [29]. Interestingly, the size of CTCs was very similar to the rest of leukocytes that were enriched in the CellSearch cartridge, implying that a size-based technique would miss many CTCs that fall in the same size range as leukocytes. 

On the other hand, CTCs and leukocytes found on the microsieves after whole blood filtration had either lost most of their distinct surface features or they had experienced major deterioration and membrane rupture that has not been reported before. The cell deformation and stretching could be attributed to the applied pressure of 100 mbar, the distance from the neighboring pores (9 μm in vertical and diagonal axes and 19 μm in horizontal axis), and the pore size of 5 μm. Cote et al. have also highlighted the importance of a strong cell fixative before blood filtering [15]. The provider of the used microsieves, VyCAP, suggests that blood samples should be collected in Transfix preservative to ensure improved cell morphology, implying that that CellSave used here for one to two day(s) is probably inadequate for optimal cell preservation. Lower pressure, more pores, and longer distances between them are parameters that could also improve the final cell morphology. Their further investigation is highly recommended mainly in cases where CTCs are isolated from EDTA blood and need to remain viable or be expanded for studies of their secretome or drug screening. 

It is noteworthy to mention that in case of the CellSearch isolated CTCs, when ferrofluid was not covering the cell surface, some granular structures could be observed on the CTC surfaces, as indicated by arrows in Figure 4 and Figure S1. A speckled CK pattern in the respective immunofluorescence images would confirm the reorganization of their cytoskeleton and their early stage of apoptosis [17,30,31], whereas a fragmented nucleus would even indicate advanced apoptosis [30]. However, in many CTCs where the punctuated CK is clearly distinguishable, the cells are fully covered by the ferrofluid, hindering the observation of blebs on the cells. In other cases, the cells, even if they had a homogeneous cytokeratin staining, had spheroid globules on their surfaces, raising the question of membrane blebbing preceding the reorganization of the keratin filament network. Alternatively, these globules could be secreted tdEVs or EVs that ended up by coincidence at the same location (Panels B,C of Figure S1). 

Interestingly, the SEM imaged blebbing of CTCs found on the microsieves corresponds to the condensed CK granular structures of the respective immunofluorescence images. That suggests that the membrane blebbing during apoptosis and the speckled CK occur simultaneously and that the observed cleaved CK pattern is not only intra-cytoplasmatic, but also extra-cytoplasmatic.

We here defined tdEVs in CRPC patients as EpCAM+, DNA−, CD45−, CK+ particles obtained after centrifugation of the blood samples of patients at 800× *g* for 10 min and their further processing on the CellSearch system. As a consequence of the blood centrifugation, the majority of isolated tdEVs have a diameter above 1–2 μm. Our previous results showed that the presence of these tdEVs isolated by the CellSearch are strongly associated with the clinical outcome of CRPC patients similarly to the CTCs [18,19]. Importantly, these tdEVs are rarely found in healthy donors and, in that case, their frequencies are significantly lower compared to the respective ones in CRPC patients (median value of 8 in 16 healthy donors and median value of 116 in 84 CRPC patients) [19]. Vagner et al. [32] and Minciacchi et al. [33] have demonstrated that large oncosomes of a diameter above 1 μm can be found in the circulation of advanced prostate cancer patients, and constitute a separate subclass of tumor-derived extracellular vesicles that carry most of the circulating tumor DNA, reflecting the genetic aberrations of the tumor of origin. These large tdEVs do not express CD81 and CD63, which are common exosome markers, and they have a distinct protein cargo [33]. CK18 is one of the significantly increased proteins expressed in that class, which is also supported by our findings. Some of these tdEVs are expected to be apoptotic bodies secreted by either the CTCs undergoing apoptosis or the tumor itself. Larson et al. [17] categorized EpCAM+, CK+ events into three different categories after the inclusion of M30 staining, which binds to an epitope accessible after caspase-cleaved CK18. The three classes were “intact” CTCs, CTCs “undergoing apoptosis”, and “CTC fragments” (DAPI−, CK+, CD45−, M30+, or M30−). “CTC fragments” could nowadays be further classified to tumor-derived apoptotic bodies (DAPI−, CK+, CD45−, M30+) and tumor derived microvesicles (DAPI−, CK+, CD45−, M30−). Interestingly, no clear pattern could be observed in the different patient samples shown: One patient had only 10% of big tdEVs positive for M30, while another one had 85% of them positive for M30. 

Nevertheless, EVs have a wide size range, with the majority of them constituting the exosome subclass with a diameter below 200 nm [34,35]; hence, most of the tdEVs are supposed to end up in the plasma fraction of the patient samples, which is not processed by the CellSearch system. Processing plasma of CRPC patients with the CellSearch system could reveal what the actual proportion of smaller tdEVs is. Preliminary results (data not shown) indicate that isolation of tdEVs from plasma of patients is indeed feasible using the CellSearch, but further investigation is needed. It should be taken into consideration that the smaller size tdEV populations may express very low amounts or even no EpCAM on their membranes depending on their biogenesis. Ferrofluid conjugated with multiple antibodies recognizing more than one tumor- or epithelial- specific surface biomarkers (e.g., αEpCAM together with αCaveolin-1 and αPSMA) and incubated in the plasma of patient samples and downstream characterization of the isolated EVs could provide higher tdEV capture yields and more insights about the cells of origin. There are some SEM images of EVs in the literature [36,37]; however, the identity of the depicted particles is always doubtful since no other correlative technique is being used to confirm the chemical composition or the surface marker expression of the imaged EVs in a single level.

Herein, the fluorescence imaging of tdEVs with αCK-PE staining and their capture by αEpCAM ferrofluid, which are both epithelial specific markers, with CK being expressed in the interior of epithelial cells and EpCAM on their surface, confirm their epithelial/tumor origin. Particles of a similar size as the ones shown in Figure 6, captured by the αEpCAM ferrofluid, were also found, but they were negative for CK, CD45, and DNA (Figure S3), and were not detected by the CellTracks Analyzer II. Further investigation using additional antibodies against generic membrane markers, like wheat germ agglutinin, or cell-specific antigens, such as HER2 (breast), PSMA (prostate), CD16 (leukocytes), or CD61 (platelets), could reveal the origin of these particles as already demonstrated by de Wit and Zeune [38]. 

## 4. Materials and Methods

### 4.1. Cell Culture

PC3 and LNCaP prostate cancer cell lines were provided by the ATCC. 10,000 cells/cm^2^ of PC3 and LNCaP prostate cancer cell lines were cultured in DMEM culture medium supplemented with 100 Units/mL penicillin and 100 μg/mL streptomycin (Lonza, cat # 16-602E) and 10% (*v*/*v*) FBS (Sigma-Aldrich Chemie BV, cat # F7524, Zwijndrecht, The Netherlands). When cells reached 80% confluence, they were washed with sterile Phosphate Buffered Saline (PBS) and trypsinized using 0.05% trypsin EDTA (Gibco, cat # 25300-062) for 2–3 min at 37 °C. After cell detachment, the trypsin was deactivated by an excess of FBS-containing culture medium. The cells were mixed to a homogenous cell suspension and kept in ice until further processing and spiking in blood samples.

### 4.2. CRPC Patient and Healthy Donor Blood Samples

10 mL of CellSave blood samples from 12 anonymous healthy donors (HDs) were obtained after written informed consent from the TNW-ECTM-donor services (University of Twente, Enschede, The Netherlands) and were used as positive controls after spiking prostate cancer cells from tumor cell lines. More specifically, 6 samples were used to spike 300–500 PC3 and 6 more to spike 300–500 LNCaP cells, and used to develop and optimize the preparation protocol after CTC isolation for SEM imaging. Half of the samples from each condition were processed by the CellSearch system (Menarini, Huntingdon Valley, PA, USA) and the other half by filtration through 5 μm microsieves (VyCAP, Deventer, The Netherlands) within 2 days from the drawing day. 

CellSave blood samples of 9 patients were provided by the Royal Marsden Hospital and processed within 2–3 days from the drawing day with either the CellSearch system (5 samples) or filtered through 5 μm pore microsieves (4 samples). The trial CCR2472 was approved by the Royal Marsden Research Ethics Committee (ethics reference number: 04/Q0801/60). Patients had metastatic prostate cancer progressing despite castrate levels of testosterone after histological confirmation and had provided written informed consent to trial protocols approved by the institutional review boards at each participating center. 

### 4.3. Immunomagnetic CTC and tdEV Isolation

7.5 mL of blood samples were centrifuged at 800× *g* for 10 min without breaks. The plasma (x volume) 1 cm above the buffy coat was removed and (5.5 + x) mL of dilution buffer was added to the samples, mixed gently, and centrifuged again at 800× *g* for 10 min without breaks. The EpCAM+ CTCs and EpCAM+ tdEVs were positively selected by immunomagnetic beads (ferrofluid) conjugated with αEpCAM from 7.5 mL of blood of cancer patients using the CTC kit in the CellSearch system as previously described [39]. Briefly, the centrifuged samples were added in the fully automated CellTracks Autoprep. Once the interface between the red blood cells and the remaining diluted plasma was detected, the diluted plasma was discarded. aEpCAM ferrofluid of the CTC kit together with a capture enhancement reagent were added in the samples. Cells within the tube were surrounded by 3 magnets moving backwards and forwards to increase the collisions between the ferrofluid and the cells. When the bound to ferrofluid cells were accumulated at the bottom of the tube, ferrofluid-free cells were discarded. The enriched objects (CTCs, leukocytes, and EVs) were permeabilized with saponin and incubated with the staining solution of the CTC kit containing the nuclear dye DAPI, antibodies against epithelial-specific cytokeratins αCK-PE (clones C11 and A53-B/A2), and an antibody against the leukocyte-specific cluster of differentiation CD45, αCD45-APC. Following some incubation and washing steps, cells were fixed and loaded into a cartridge placed within a magnest for their imaging using a semi-automatic 10× objective fluorescence microscope, the CellTracks Analyzer II. The CellSearch definition of a CTC was to have a cell-like morphology, have a nucleus (DAPI+), express cytokeratin (CK+), have a size bigger than 4 μm, and do not express CD45 (CD45−). tdEVs were defined as particles expressing only cytokeratin (DNA−, CK+, CD45−) as previously described [19]. 

### 4.4. Size-Based CTC Isolation

7.5–10 mL of CellSave blood samples of CRPC patients and healthy donors spiked with prostate cancer cell lines were filtered through 5 μm microsieves provided by VyCAP. 100 mbar was applied to the filters using a pump unit. Once the whole volume of blood was filtered, cells were washed once with 0.15% (*w*/*v*) saponin in 1% (*w*/*v*) BSA in PBS. Permeabilization using 0.15% (*w*/*v*) saponin in 1% (*w*/*v*) BSA in PBS for 15 min at room temperature (RT) was followed. A staining solution containing 1 μg/mL αCKs (clone C11) -PE (Veridex, cat #PN6030), 2 μg/mL α-Pan CK-efluor 570 (eBioscience, cat #41-9003-80, clone AE1/AE3), 4 μg/mL Hoechst 33342 (Invitrogen, cat # H3570, Burlington, ON, Canada), and 4 μL of αCD45-PerCP (Invitrogen, cat #MHCD4531) in a final volume of 50 μL of 0.05% (*w*/*v*) saponin in 1% BSA in PBS was applied in each sample for 15 min at 37 °C in a humidified environment. Two washing steps were followed. The cells were finally fixed with 1% (*v*/*v*) formaldehyde in PBS for 10 min at RT. One more washing step using 1% (*w*/*v*) BSA in PBS was used. The cells on the microsieves were further prepared as described in Section 4.5 before being imaged using a 20× objective fluorescence microscope. 

### 4.5. Specimen Preparation for Scanning Electron Microscopy (SEM)

Isolated objects in the CellSearch cartridges or on the microsieves were fixed with 4% formaldehyde for 15 min at RT to better retain their morphology in the following preparation steps. Subsequent dehydration was required to exchange the high water- content of cells and EVs with ethanol (that has lower surface tension to air than water to air) and hinder cell deformation and corruption when in the vacuum chamber of the SEM. Therefore, gradual dehydration from 70% (*v*/*v*) ethanol in milliQ up to 100% ethanol with a 10% (*v*/*v*) ethanol concentration increment step (70%, 80%, 90%, 100% (*v*/*v*) ethanol) took place at RT. Each dehydration step lasted for 5–10 min at RT. After 100% ethanol, 1:1 ethanol:hexamethyldisilazane (HMDS) was added to the cells for 3–5 min at RT followed by a final step of 3–5 min incubation in HMDS and air drying. 

In the case of CellSearch cartridges, the glass slides were removed from the cartridges. All specimens were left overnight at RT for the HDMS residual to be evaporated. 

Since SEM is based on the electrons passing through the material of interest, an electrically conductive sample is required. Cells and tdEVs as well as glass slide and microsieve substrates have a low electron density, resulting in reduced electron scattering and low quality images, sample charging, damage and carbon deposition; hence, a metal coating is required. Herein, 10 nm gold coating was applied using the JEOL JFC-1300 Auto Fine Coater (10 times of 1 nm thickness gold was applied using 10 mA for 30 s).

### 4.6. Scanning Electron Microscopy (SEM)

The dehydrated and dried specimens were mounted on silicon holders with a both sided glue carbon tab and placed in the vacuum chamber of SEM (JEOL JSM-6610LV) for further analysis. A region of interest (ROI) containing single or multiple cells was first localized with SEM using relatively low magnification (~350×). The SEM was operated at 4–5 kV acceleration voltage using secondary electron (SE) detection. Once the regions of interest were relocated following the cell patterns of the respective fluorescence images, SEM images of CTCs and tdEVs were recorded using a higher magnification (3000× to 10,000×). 

## 5. Conclusions

In conclusion, our findings suggest that CTCs isolated by the CellSearch system retain their cell morphology and have different shapes and sizes. However, the coverage of most of their surfaces by the αEpCAM ferrofluid prevents their detailed morphological characterization, while at the same time, raises questions about the staining efficiency of additional surface markers expressed in low levels mainly after CTC isolation. Conversely, CTCs isolated purely by filtration through 5 μm microsieves experienced major cell deformation due to several factors, including the applied pressure, limited number of pores, and close pore proximity. In studies that CTCs need to be viable after their isolation and get expanded, e.g., studies of CTC secretome, drug efficiency testing, or for understanding the underlying biology of tumor growth and mutational status, the CTC profile kit is available from the CellSearch system, where CTCs can be immunomagnetically isolated from EDTA blood samples without being further fixed, permeabilized and stained. In the case of filtration, less harsh handling is required to be further evaluated, such as lower applied pressure, or incorporated microsieves within microfluidics to ensure CTC viability.

Finally, the SEM imaging of the CellSearch cartridges also allowed imaging of EpCAM+, CK+, CD45−, DNA− tdEVs isolated from the blood cell fraction of the patient samples, confirming their three-dimensional shape and that these are not just cell membrane fragments or antibody artefacts. The existence of particles of similar size and shape that were not detected by immunofluorescence imaging because of the lack of nucleus and CK, CD45 expression requires further investigation to reveal their origin.

## Supplementary Materials

The following are available online at http://www.mdpi.com/2072-6694/10/11/416/s1, Figure S1: Gallery of additional correlated fluorescence and SEM images of CTCs of a CRPC patient isolated by the CellSearch system, Figure S2: Gallery of additional correlated fluorescence and SEM images of CTCs of a CRPC patient isolated by whole blood filtration using 5 μm microsieves, Figure S3: SEM imaging of a CTC and an undetected by fluorescence particle isolated by the CellSearch from the blood of a CRPC patient, Figure S4: Correlated fluorescence and SEM images of spiked 1. PC3 and 2. LNCaP cells isolated by A. the CellSearch system or B. whole blood filtration using 5 μm microsieves, Figure S5: SEM images of 15 leukocytes (out of 85 that were SEM imaged) isolated by the CellSearch from blood samples of 3 cancer patients, Figure S6: Comparison of A. manufacturer’s (VyCAP) and B. followed protocol for the permeabilization and fixation of the isolated cells/CTCs through 5 μm pore microsieves.
