# Supplementary material for: Scanning Electron Microscopy of Circulating Tumor Cells and Tumor-Derived Extracellular Vesicles
Source: Cancers (Basel). 2018 Oct 31;10(11):416. doi: 10.3390/cancers10110416 (PMC6266016; doi:10.3390/cancers10110416)
Supplement: Supplementary file 1 [file cancers-10-00416-s001.pdf]

cancers

Supplementary material

Scanning Electron Microscopy of Circulating Tumor Cells and Tumor-Derived Extracellular Vesicles

Afroditi Nanou, Mateus Crespo, Penny Flohr, Johann S. De Bono and Leon W.M.M. Terstappen

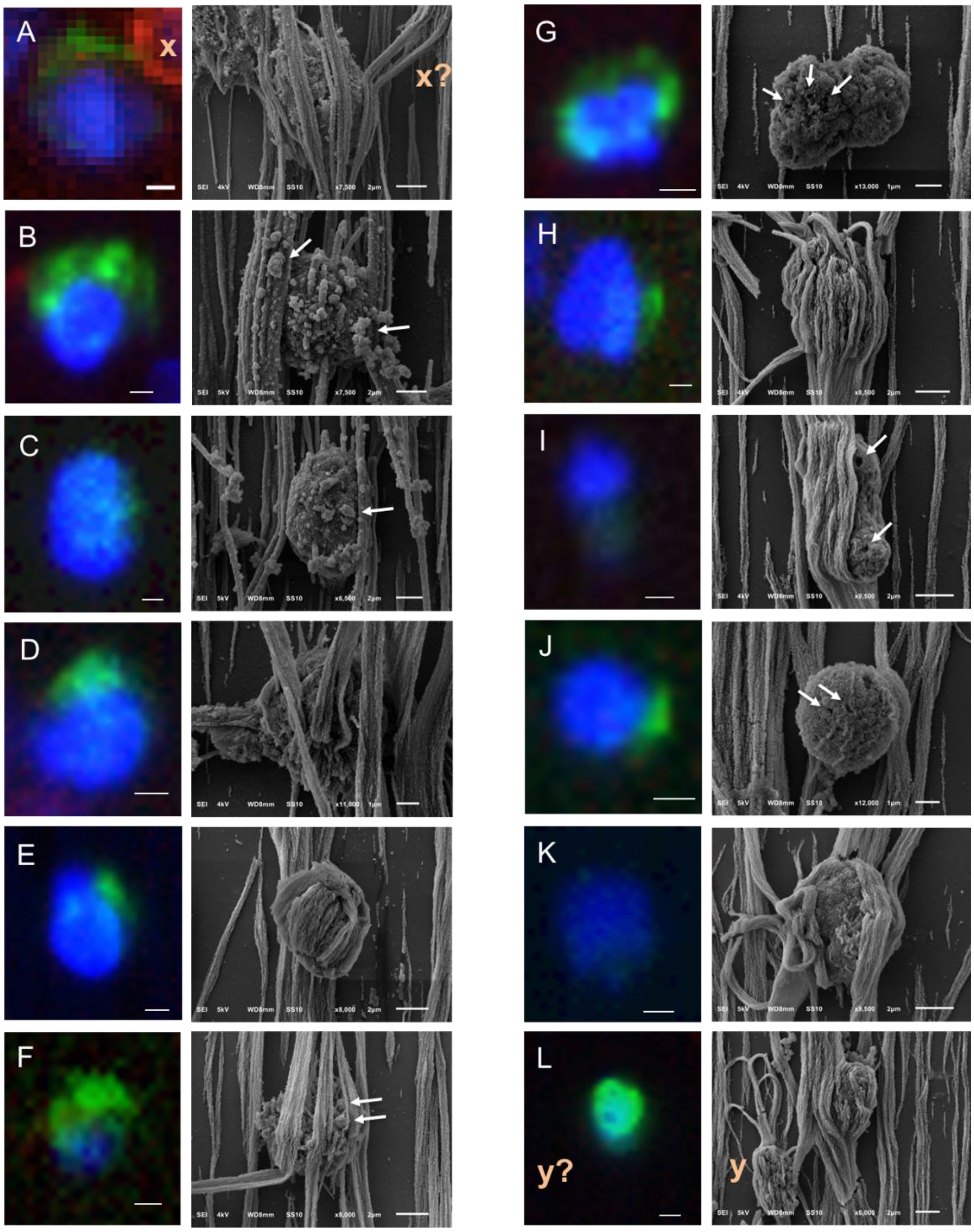

Figure S1. Gallery of additional correlated fluorescence and SEM images of CTCs of a CRPC patient isolated by the CellSearch system. Nucleus (DNA) is represented by blue, CD45 by red and CK by green. Arrows are pointing at vesicles found beneath (Panel **F**) or on top (Panels **B**,**C**) of the ferrofluid covering the CTCs. Some CTCs appear to have sponge-like surfaces with visible holes as pointed by arrows (Panels **G**,**I**,**J**). Few cells (like "x" in Panel **A**) could not be relocated by SEM, because they were lost during the dehydration/drying procedure. In other cases, additional objects (like "y" in Panel **L**) that were invisible by fluorescence could be visualized by SEM. Scale bars of fluorescence images indicate 2 μm.

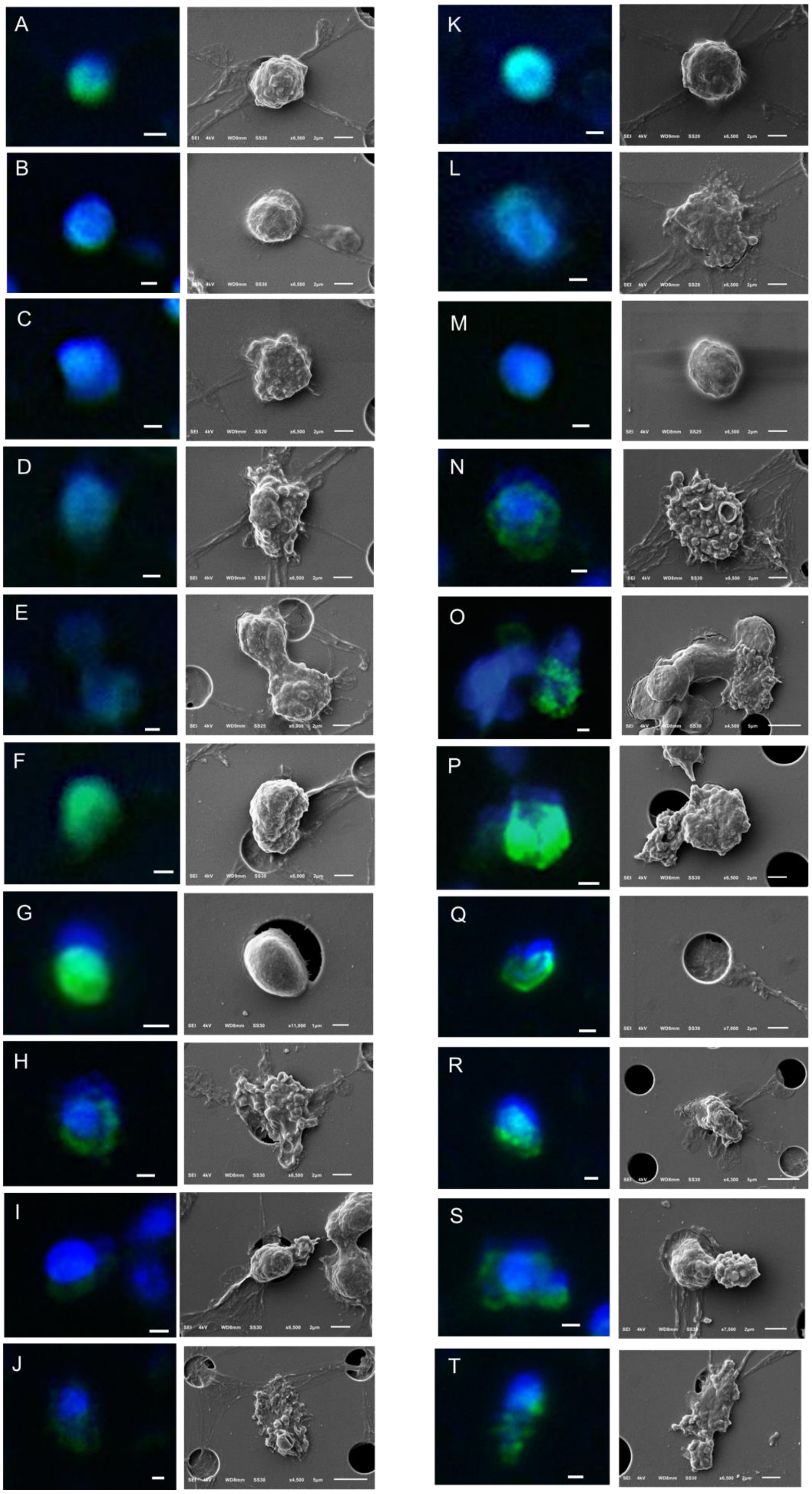

Figure S2. Gallery of additional correlated fluorescence and SEM images of CTCs of a CRPC patient isolated by whole blood filtration using 5 µm microsieves. Nucleus (DNA) is represented by blue and CK by green. CTCs were found on (Panels **A**–**D**,**F**–**K**,**M**–**P**,**R**–**T**), inside (Panels **J**,**L**,**Q**) and in between (Panel **E**) the pores. Apoptotic CTCs with punctuated CK pattern appeared to have blebs on their surfaces (Panels **H**,**J**,**N**,**O**,**T**). Scale bars of fluorescence images indicate 2 µm.

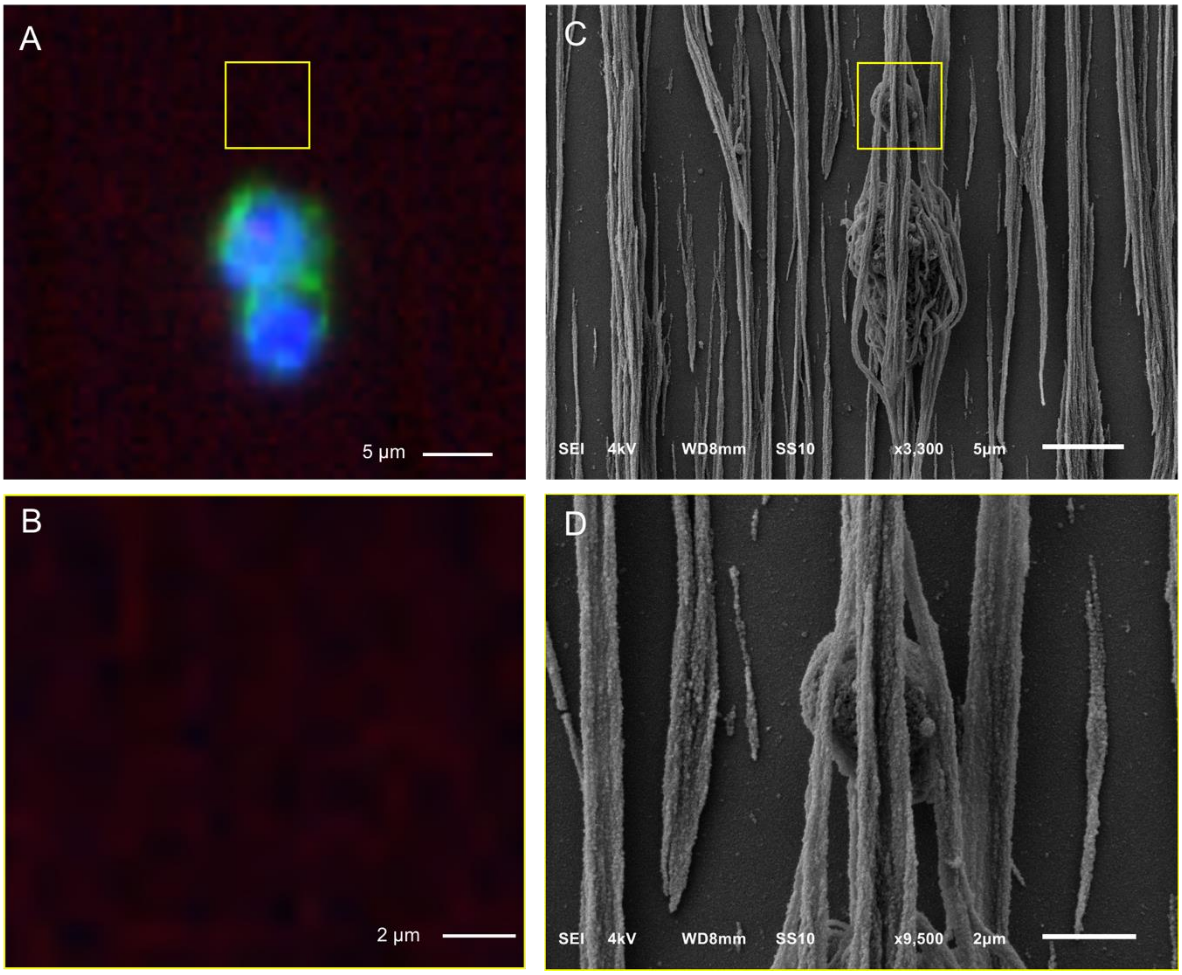

Figure S3. SEM imaging (Panel **C**) of a CTC and an undetected by fluorescence (Panel **A**) particle isolated by the CellSearch from the blood of a CRPC patient. The particle has a similar size to tdEV, but does not express CK (represented by green). Nucleus (DNA) is represented by blue. Magnified fluorescence and SEM images of the enclosed by the yellow square particle are shown in panels **B**,**D** respectively.

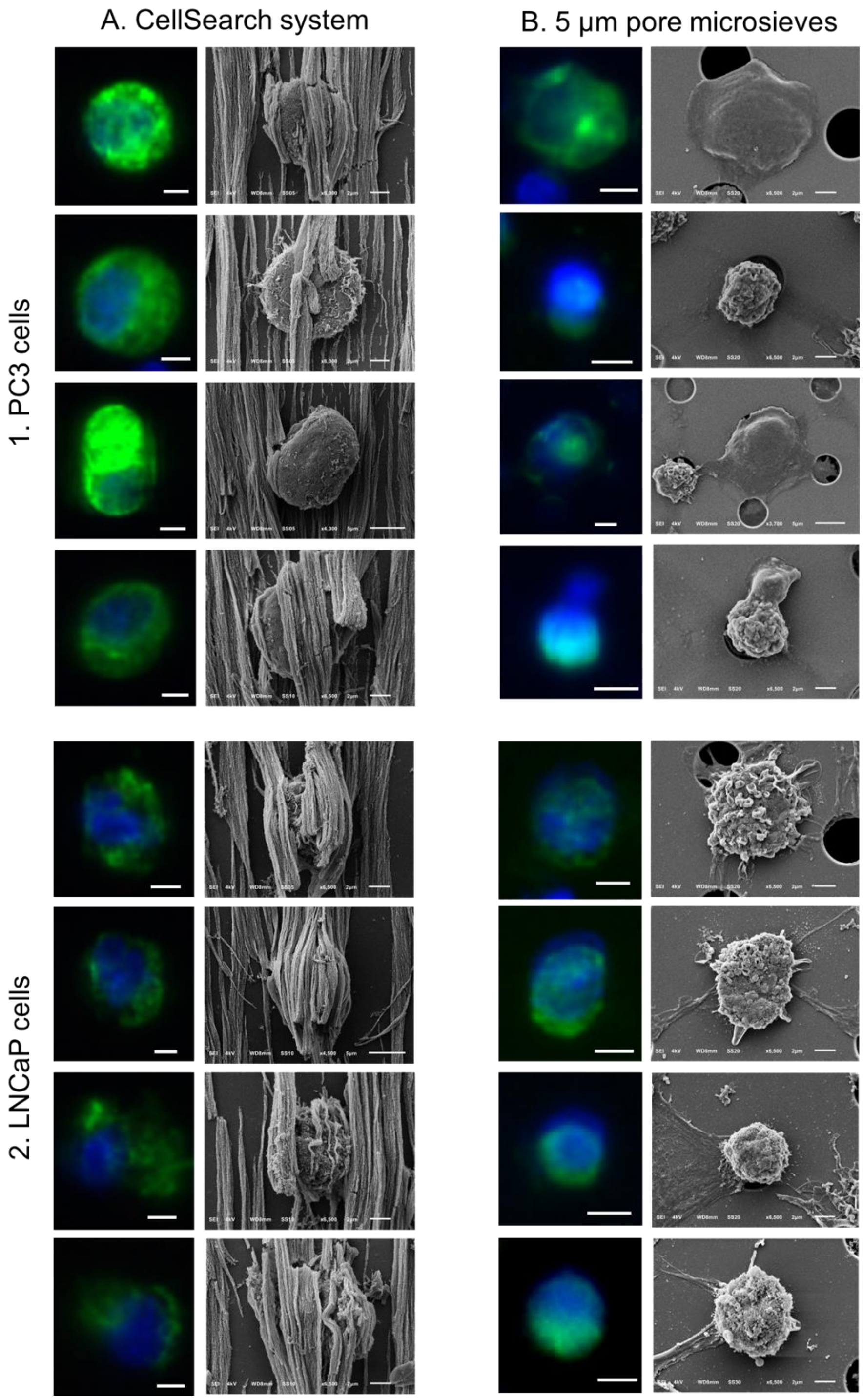

Figure S4. Correlated fluorescence and SEM images of spiked 1. PC3 and 2. LNCaP cells isolated by the CellSearch system (Panel **A**) or whole blood filtration using 5 µm microsieves (Panel **B**). Nucleus (DNA) is represented by blue and CK by green. 20–30 different cells from each condition were SEM imaged, but only 4 are presented here. Scale bars indicate 4 µm.

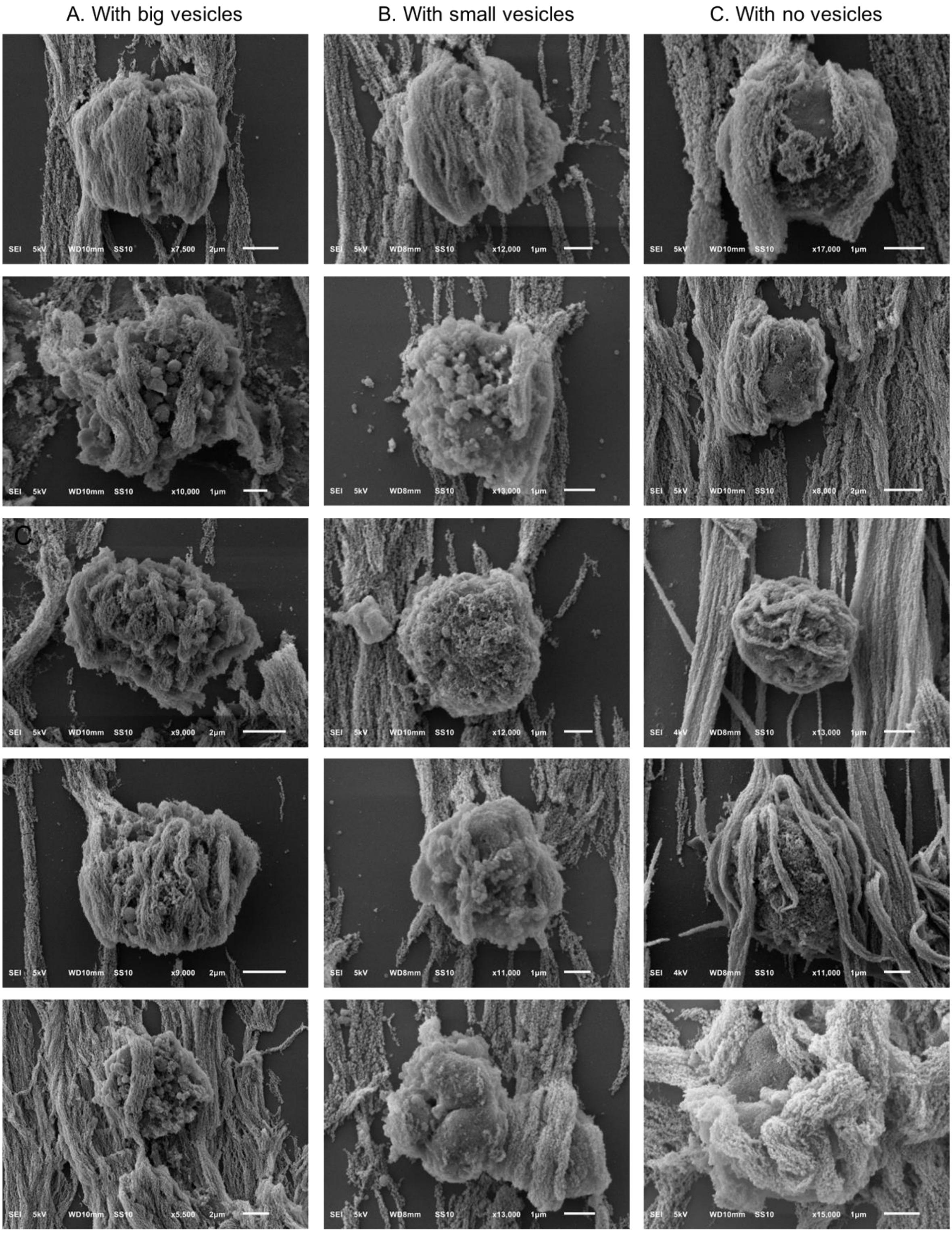

Figure S5. SEM images of 15 leukocytes (out of 85 that were SEM imaged) isolated by the CellSearch from blood samples of 3 cancer patients. 15% (13/85) of the isolated leukocytes that were SEM imaged bore big vesicles (Panel **A**) on their surfaces that were not observable in case of CTCs. 55% (47/85) had smaller vesicles (Panel **B**) on their surfaces and 30% (25/85) had no vesicles (Panel **C**) at all on their surfaces.

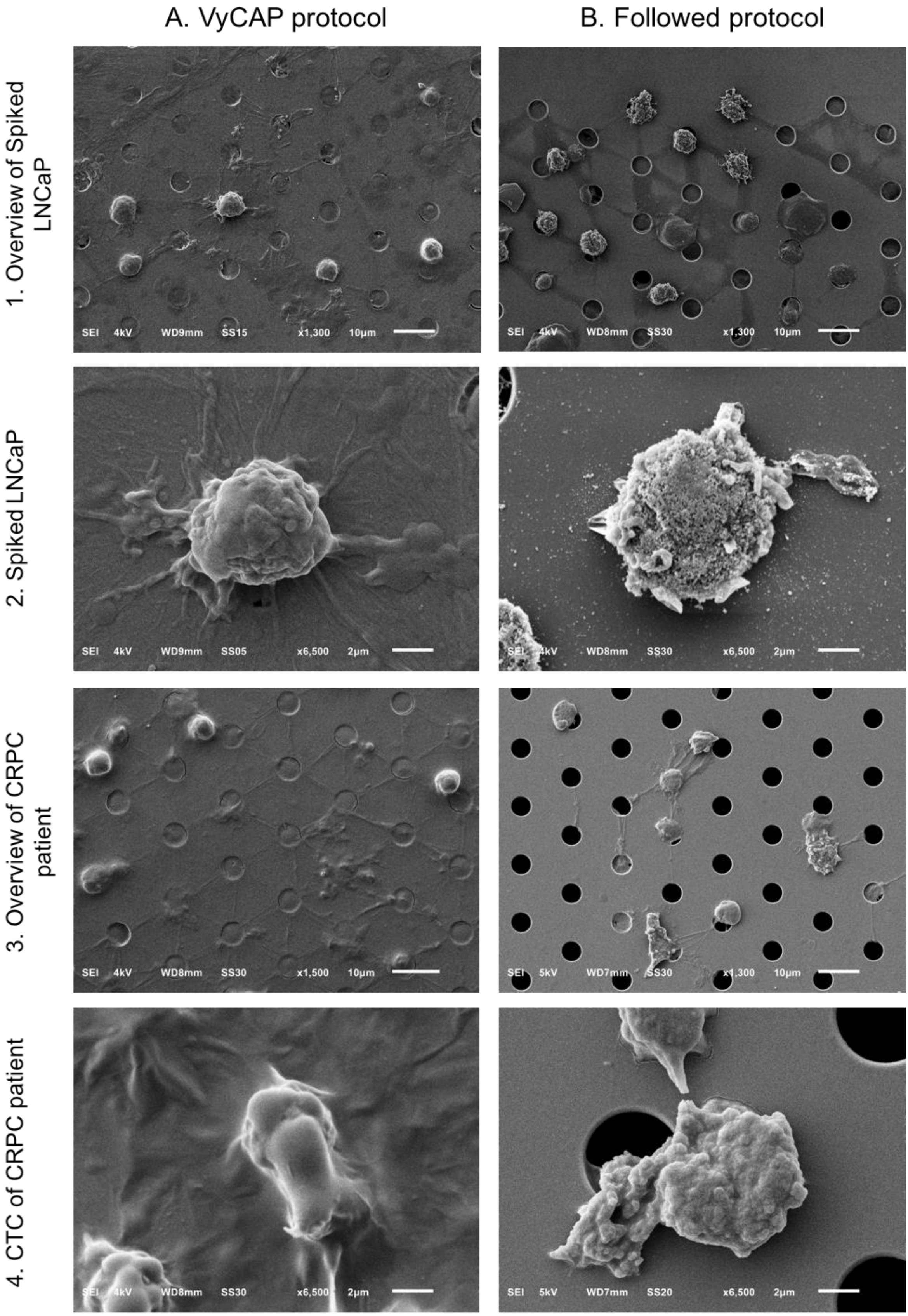

Figure S6. Comparison of **A**. manufacturer's (VyCAP) and **B**. followed protocol for the permeabilization and fixation of the isolated cells/CTCs through 5 µm pore microsieves. Both protocols were applied using spiked samples (Panels **1**,**2**) and CRPC patient (Panels **3**,**4**) samples. VyCAP protocol resulted in higher cell background during the SEM imaging that hindered the imaging of individual CTCs.
